# Smart Immunosensors for Point-of-Care Serological Tests Aimed at Assessing Natural or Vaccine-Induced SARS-CoV-2 Immunity

**DOI:** 10.3390/s22145463

**Published:** 2022-07-21

**Authors:** Simone Fortunati, Marco Giannetto, Chiara Giliberti, Angelo Bolchi, Davide Ferrari, Massimo Locatelli, Valentina Bianchi, Andrea Boni, Ilaria De Munari, Maria Careri

**Affiliations:** 1 Dipartimento di Scienze Chimiche, della Vita e della Sostenibilità Ambientale, Università di Parma, Parco Area delle Scienze 17/A, 43124 Parma, Italy; simone.fortunati@unipr.it (S.F.); chiara.giliberti@unipr.it (C.G.); angelo.bolchi@unipr.it (A.B.); davide.ferrari@unipr.it (D.F.); 2 IRCCS Ospedale San Raffaele, Via Olgettina 60, 20132 Milano, Italy; locatelli.massimo@hsr.it; 3 Dipartimento di Ingegneria e Architettura, Università di Parma, Parco Area delle Scienze 181/A, 43124 Parma, Italy; valentina.bianchi@unipr.it (V.B.); andrea.boni@unipr.it (A.B.); ilaria.demunari@unipr.it (I.D.M.)

**Keywords:** SARS-CoV-2, COVID-19, serological test, immunosensor, point-of-care testing, IoT-Wi-Fi

## Abstract

Innovative and highly performing smart voltammetric immunosensors for rapid and effective serological tests aimed at the determination of SARS-CoV-2 antibodies were developed and validated in human serum matrix. Two immunosensors were developed for the determination of immunoglobulins directed against either the nucleocapsid or the spike viral antigen proteins. The immunosensors were realized using disposable screen-printed electrodes modified with nanostructured materials for the immobilization of the antigens. Fast quantitative detection was achieved, with analysis duration being around 1 h. Signal readout was carried out through a smart, compact and battery-powered potentiostat, based on a Wi-Fi protocol and devised for the Internet of Things (IoT) paradigm. This device is used for the acquisition, storage and sharing of clinical data. Outstanding immunosensors’ sensitivity, specificity and accuracy (100%) were assessed, according to the diagnostic guidelines for epidemiological data. The overall performance of the sensing devices, combined with the portability of the IoT-based device, enables their suitability as a high-throughput diagnostic tool. Both of the immunosensors were validated using clinical human serum specimens from SARS-CoV-2 infected patients, provided by IRCCS Ospedale San Raffaele.

## 1. Introduction

The SARS-CoV-2 virus, first identified in December 2019 in Wuhan, China, caused a globally diffused acute respiratory disease called COVID-19, which was declared a pandemic by the World Health Organization (WHO) in March 2020 [1]. As of 1 June 2022, more than 520 million people were infected globally and over six million died as a result of infection [2]. Several diagnostic tools were developed since the appearance of COVID-19. The frontline approaches for the early diagnosis of the SARS-CoV-2 infection, aimed at containing the outbreaks, were represented by the detection of viral RNA or viral antigens in the respiratory mucosa by molecular and antigenic swab tests; however, the serological determination of antibodies targeting the viral proteins provides information about previous or current infection, as well as information about the vaccination response [3,4]. Exceptional research efforts led to the rapid development of several vaccines which have already been distributed to a large part of the general population [5]. The health and socio-economic impact of the spread of the pandemic has highlighted the need for rapid and efficient analytical methods suitable for point-of-care Testing (PoCT) in non-hospital settings [6,7,8,9].

In this context, lateral flow immunoassays (LFIA), also known as immunochromatographic strip tests, which are commonly used for the rapid serological screening of SARS-CoV-2, represent a powerful tool for monitoring the immunization degree of the population. However, a qualitative conclusion (yes/no) associated with a low analytical sensitivity and poor diagnostic performance represent major drawbacks of the rapid test, which is known to be more likely to return a false-negative or false-positive compared to other methods [10]. Other COVID-19 serological testing, relying on targeted antibodies binding to SARS-CoV-2-specific antigens, include the chemiluminescence immunoassay (CLIA), enzyme-linked immunosorbent assay (ELISA) and immunofluorescence assay (IFA): recent review articles describe the advantages and disadvantages of these methods, based on detection efficiency, system costs, the convenience of operation process and the limitations of application in COVID-19 diagnosis [11,12].

Anti-SARS-CoV-2 antibodies detectable by serological screening can be directed against the nucleocapsid protein (anti-N) or the spike protein (anti-S) of SARS-CoV-2. More specifically, anti-N immunoglobulins (Ig) can only be found in previously or currently infected subjects, thus being diagnostic for SARS-CoV-2 infection [13]. In contrast, the anti-S Ig antibodies, playing a key role in the neutralizing activity of the live SARS-CoV-2 virus, can also be vaccination-induced and provide information about the vaccines’ response [14]. Electrochemical immunosensors represent a valid alternative to immunochromatographic strip tests, responding to the need for rapidity of the assay and portability of the instrumentation, also providing higher analytical sensitivity and allowing the quantification of antibodies [15].

To date, few literature studies have addressed the serological determination of anti-SARS-CoV-2 antibodies by electrochemical immunosensors [16,17,18]. Torrente-Rodrìguez et al. reported on a laser-engraved graphene-based multiplexed electrochemical platform for the ultra-rapid detection of anti-S IgM and IgG antibodies, as well as the nucleocapsid protein and the inflammatory biomarker C-reactive protein [16]. Although the multiplexed sensing device was applied to the analysis of serum and saliva samples, important parameters that affect the quantitative issues of an analytical method, such as limit of detection (LOD) and limit of quantitation (LOQ), were not assessed. In addition, the multiplexed device was not subjected to analytical validation in biological fluids of diagnostic concern, as the calibration curves were acquired in buffered saline solutions. More recently, Yakoh and coworkers developed a paper-based immunosensor for label-free determination of the Ig produced against SARS-CoV-2 [17]. The system was tested in serum specimens, but only anti-S antibodies were considered, reaching LOD values of 0.96 and 0.14 ng/mL for IgG and IgM, respectively. Among the label-free electrochemical sensors, a commercially available impedance-sensing platform, based on a 16-well plate containing electrodes pre-coated with the receptor-binding domain (RBD) of the SARS-CoV-2 spike protein, was recently developed [18]; using this approach, the rapid and accurate detection of the SARS-CoV-2 antibodies at clinically relevant concentrations was emphasized. However, the authors were not able to demonstrate the LOD of the method, due to both hardware noise and variations in sample handling. Very recently, Peng et al. developed a portable serological testing platform for the rapid electrochemical detection of the SARS-CoV-2 antibodies [19] Although the methodological approach is similar to that used in the present study, the overall analytical performance for the determination of the anti-S antibodies cannot be compared, due to different units of measurement. Once again, only the anti-S antibodies were taken into account. Furthermore, this device relies on a Bluetooth communication protocol and then requires an external device for operation. A crucial aspect is the difficulty of comparing the performance and results of different serological tests. As reported in a recent paper by Ferrari et al., comparisons are challenging even when the same antigen (S-protein) is used, because most of the currently available devices are based on different technologies, detect different types of immunoglobulins and use a variety of different S-protein targets [20]. Furthermore, the results are usually expressed in arbitrary units, making comparisons between different methods challenging. In this regard, in order to reduce inter-laboratory variation, the National Institute for Biological Standards and Control (NIBSC) and WHO adopted on 10 December 2020, an International Standard (IS) based on a pool of human plasma from eleven convalescent patients to allow the calibration of assays into Binding Antibody Units per mL (BAU/mL) [21,22]. Due to the limited amount and the difficulty to obtain aliquots of the IS, Ferrari et al. produced a working standard (WS) based on a pool of human plasma from fifty-two vaccinated individuals [20], which was calibrated against the WHO’s IS.

In such a context, the aim of the present study was to develop and validate smart voltammetric immunosensors, based on the use of nanostructure-functionalized screen-printed electrodes (SPE). Attention was paid to the nanomaterials used as an interface for the immobilization of receptor antigens, i.e., nucleocapsid protein or RBD of the spike protein, by comparing the behavior of gold nanoparticles (GNP) and/or single-walled carbon nanotubes (SWCNT) as far as the analytical characteristics of the SPEs and amplification of the current responses are concerned. The immunosensors were developed for anti-N and anti-S antibodies’ detection, with discrimination between IgG and IgM in the first case. As for the detection of the immunosorbed target antibodies, an isotype-specific secondary antibody, conjugated with alkaline phosphatase (AP) as the enzyme tag, was used for the generation of the electroactive species. Based on the different nature of the antigen against which the antibodies are directed, a qualitative method was developed for the detection of anti-N, while for the detection of anti-S, a quantitative method was developed, useful for screening the immune response of patients in the context of vaccination programs [14]. The immunosensors were subjected to validation according to the diagnostic guidelines for epidemiological data [23]. For this purpose, the aforementioned WS and real serum samples provided by the IRCCS Ospedale San Raffaele (OSR) were used, with an estimated antibody rate using a Roche Elecsys^®^ Anti-SARS-CoV-2 chemiluminescence immunoassay and expressed as (BAU)/mL.

The readout of the signal was carried out through a smart, compact and battery-powered potentiostat based on a Wi-Fi protocol and compliant with the Internet of Things (IoT) paradigm for the acquisition, storage and sharing of clinical data [24,25].

To our knowledge, this is the first electronic device which, combined with the developed immunosensors, fulfills the need for both the point-of-care diagnosis of COVID-19, allowing untrained personnel to perform laboratory diagnostics, and the screening of the immunity of the population. The speed, high portability and the ability to perform real-time analysis ensure excellent application potential in screening programs to meet the growing demand for fast, easy-to-use and accurate diagnostics to facilitate COVID-19 testing outside of the laboratory settings.

## 2. Materials and Methods

### 2.1. Materials

Sodium chloride (NaCl), potassium chloride (KCl), potassium dihydrogen phosphate (KH_2_PO_4_), disodium hydrogen phosphate (Na_2_HPO_4_), Trizma^®^ Base, sodium hydroxide (NaOH), hydrochloric acid (37% *w*/*v*) (HCl), magnesium chloride (MgCl_2_), potassium ferrocyanide (K_4_[Fe(CN)_6_]), *N*-(3-dimethyl)-*N*′-ethylcarbodiimide hydrochloride (EDC), *N*-Hydroxysuccinimide (NHS), 4-morpholineethanesulfonic acid monohydrate (MES), Tween^®^ 20 and plasma from bovine, were purchased from Merck (Milan, Italy). Sars-CoV-2 Nucleocapsid His T protein, goat anti-human IgG conjugated with alkaline phosphatase (Anti-Hu-IgG-AP) and goat anti-human IgM conjugated with alkaline phosphatase (Anti-Hu-IgM-AP) were purchased from Thermo Fisher Scientific (Milan, Italy). Sars-CoV-2 Nucleocapsid antibody IgG (anti-N-IgG) and IgM (anti-N-IgM), Sars-CoV-2 Spike protein S1, SarsCoV-2 Spike S1 IgG (anti-S1-IgG) and IgM (anti-S1-IgM) antibodies, Sars-CoV-2 Spike protein RBD mFc Tag, MonoRab™ AntiMouse IgG (RAM) were purchased from GenScript (Leiden, The Netherlands). Hydroquinone diphosphate (HQDP), SWCNT-SPEs (DropSens DRP-110SWCNT), GNP-SPEs (DropSens DRP-110GNP) and SWCNT/GNP-SPEs (DropSens DRP-110CNT-GNP) were purchased from Metrohm Italiana s.r.l. (Origgio, Italy).

### 2.2. Buffer Composition

The phosphate Buffer Saline (PBS) at pH = 7.4 was prepared in distilled water with the following composition: 137 mM NaCl; 2.7 mM KCl; 1.2 mM KH_2_PO_4_; 8 mM Na_2_HPO_4_. The PBS-T was prepared by the addition of the surfactant Tween^®^ 20 to PBS to reach a final concentration of 0.05% (*w*/*v*). TRIS buffer at pH = 7.4 and reading buffer at pH = 9.8 were prepared in distilled water with the following composition: 0.1 M Trizma Base; 0.02 M MgCl_2_. The pH was adjusted with 1M HCl. TRIS-T was prepared by addition of the surfactant Tween^®^ 20 to TRIS to reach a final concentration of 0.05% (*w*/*v*). The MES buffer was prepared by dissolving 1.06 g of MES in distilled water and adjusting the pH with NaOH to 5.0.

### 2.3. Equipment for Data Acquisition

As for the characterization of the functionalized SPEs, cyclic voltammetry (CV) experiments were carried out with a benchtop potentiostat, Autolab PGSTAT 204 (Metrohm Italiana s.r.l., Origgio, Italy). The CV measurements were carried out using 5 mM potassium ferrocyanide as a redox probe, dissolved in 0.1 M KCl with a scan rate of 100 mV/s in a potential window ranging from −0.3 to 0.6 V.

**Figure 1:**
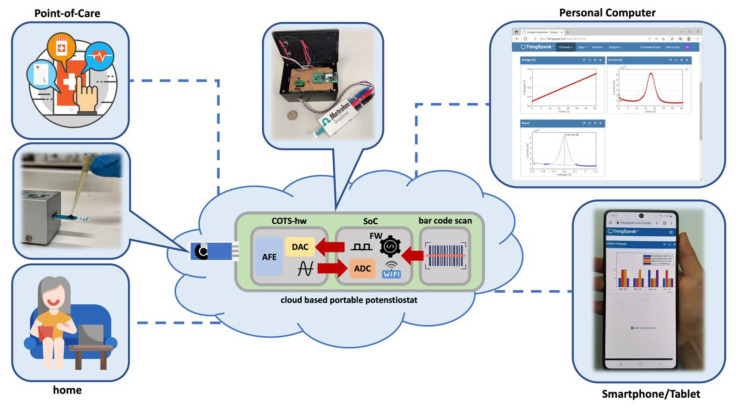
Architecture of the wireless portable potentiostat and PoCT process.

As for the readout of the immunosensors’ analytical response, the amperometric signal was generated as a result of the formation of the immunocomplex between the target and secondary antibodies, which are labelled with AP. The enzyme processes the HQDP substrate, converting it to hydroquinone (HQ), which in turn is electrochemically oxidized to quinone. The differential pulse voltammetry (DPV) current peak intensities are related to the level of the target antibodies, estimated by the serological screening test. The readout step was carried out by drop-casting 50 µL of a 1 mg/mL solution of HQDP dissolved in the reading buffer performing a DPV measurement after 150 s incubation of the enzyme substrate. A wireless portable potentiostat devised for PoCT or home testing was used for the data acquisition. The prototype device was illustrated in a previous paper [26]. The system architecture is depicted in Figure 1 and encompasses, in an IoT compliant fashion, a compact, battery-powered, hardware device for the edge computing with a cloud ecosystem to improve the storage possibilities and processing capabilities for the implementation of complex functions. The cloud is connected to a web service accessible through common web browsers available on PCs or mobile devices, providing user interaction and visualization in a simple and intuitive way, without the need to install custom apps or software.

The hardware device was designed to perform either qualitative [27], or quantitative analyses [28]. Both of the analyses require a configuration phase to set the electrical parameters of the device (e.g., cell conditioning voltage) and the implementation of a suitable calibration function for the processing of the measurement data. The system is configured only via the dedicated web interface, whereas the calibration can be performed either onboard or by exploiting the computing power of the cloud. Once a calibration procedure is complete, the obtained parameters are stored on the cloud and made available for subsequent download. This solution allows the device to easily switch between configurations, without having to be re-calibrated each time a different analysis has to be performed. This behavior increases the system flexibility, since the same compact hardware device can be moved to different locations and perform different types of analyses. The hardware device includes an analog section (COTS-hw in Figure 1) designed for exploiting COTS (Commercial-Off-The-Shelf) components and a board assembled with a CC3200 System-on-Chip (SoC) from Texas Instruments, including an Arm Cortex M4 MCU and a Wi-Fi transceiver. The former implements the cell conditioning and signal amplification functions, while the latter is dedicated to data processing and wireless communication. Furthermore, the system is equipped with a bar code optical scanner (Newland EM3080-W) for user identification via his/her health card before proceeding with the serological test. During the signal acquisition, according to the DPV method, an Analog Front-End (AFE) circuit generates a suitable voltage waveform (V_bias_) between the working and the reference electrodes (WE and RE, respectively) of the electrochemical cell. The signal conditioning circuit is based on a pair of 16-bit Digital to Analog Converters (DACs) and features voltage accuracy, which affects the V_bias_ waveform similar to bench-top instruments [26], acquiring a total of 342 measurement points. In this work, a potential scan ranging from −0.5 to 0.3 V was applied to the electrochemical cell to perform the current measurements. A bias voltage V_bias_ of −0.5 V is forced for cell preconditioning and, after 30 s, the DPV scan input is sent to the cell. The high and low values are progressively increased with 5 mV steps between successive periods. Under these conditions, a scan rate of 25 mV/s was applied, based on an optimized DPV waveform assessed in our previous studies [24,25] The output voltage is then processed onboard to calculate the current peak related to the target analyte concentration. The implemented algorithm performs baseline correction to detect the current peaks, even in the presence of baseline drift [29]. Based on the calibration parameters downloaded from the cloud, the device can perform qualitative analysis providing a positive or negative result, or quantitative analysis measuring the concentration of the target analyte. The possibility of performing onboard data processing allows the device to operate even in areas not covered by a Wi-Fi connection. Once a Wi-Fi link is available, the data are transmitted to the cloud for storage and visualization.

This architecture also ensures great flexibility: indeed, the onboard elaboration can be replaced with the on-cloud processing, given the particular application and the requirements in terms of power consumption and the computational resources needed.

In Figure 1, in the PC view the case of an on-cloud processing of a voltammogram with the peak search process is shown, whereas in the smartphone view, an example of semiquantitative analyses is depicted for the detection of positive and negative samples.

### 2.4. Serum Specimens

The blood samples from either SARS-CoV-2 infected or vaccinated subjects were from the Ospedale San Raffaele (OSR), Milan, Italy. The vaccinated subjects were healthcare professionals belonging to the OSR who were offered the Comirnaty mRNA BNT162b2 vaccine within the first two months of 2021. A set of pre-pandemic sera (i.e., withdrawn during 2018 and stored at the OSR biobank) were used as the negative samples. The subjects were part of the Covidiagnostix study approved by the Institutional Ethical Review Boards (CE:199/INT/2020). The blood samples from the vaccinated subjects were withdrawn approximately 21 days after receiving the second dose [30]. The WS used for calibration was prepared by pooling 52 samples from vaccinated subjects, as described in Ferrari et al. [20].

### 2.5. Anti-N Immunosensor Fabrication

The following procedure was adopted for the immunosensors performed on both the SWCNT/GNP-SPEs and GNP-SPEs. The nucleocapsid protein was diluted in PBS to reach a concentration of 15 µg/mL and 25 µL of this solution was drop-casted overnight on the SPEs at +4 °C. After chemisorption of the receptor antigen on GNPs occurred, the SPEs were thoroughly rinsed with PBS-T, followed by PBS. To avoid non-specific binding, a blocking step was carried out, by incubating a 25 µL drop of plasma from bovine at room temperature for 30 min. Finally, a washing step was carried out by rinsing the SPE with TRIS-T followed by TRIS. For the analysis of target antibody standards, a solution containing (i) anti-N standards at the selected concentration in TRIS, (ii) 1:50 diluted human serum and (iii) 10 µg/mL Anti-Hu-IgG-AP or Anti-Hu-IgM-AP was prepared. For the analysis of the serum specimens containing anti-N, the sample was diluted by a 1:50 factor in a TRIS solution containing 10 µg/mL alkaline phosphatase-conjugated secondary antibody (anti-Hu-AP). The mixtures containing either ant-N standards or serum specimens were drop-casted for 1 h on the surface of the modified SPE. At the end of the incubation, a washing step was performed with TRIS-T followed by TRIS. Subsequently, the voltammetric readout was carried out.

### 2.6. Anti-S Immunosensor Fabrication

The carboxylic functional groups present on the CNTs were activated for the coupling reaction by drop-casting of 50 µL of a solution of 0.2 M EDC and 0.05 M NHS in MES buffer for 30 min, followed by thorough rinsing with distilled water. Subsequently, a RAM secondary antibody solution diluted in PBS to a final concentration of 20 µg/mL was incubated on the SPE surface overnight at +4 °C. After incubation, a washing step was performed with PBS-T followed by PBS. To perform receptor immobilization, 25 µL of a 3.75 µg/mL solution of RBD-Fc was drop-casted and left for 1 h, followed by a washing step with PBS-T and PBS. Finally, the SPE surface was treated by incubating 25 µL drop of plasma from bovine for 30 min to block potential non-specific interactions. A final washing step was carried out by rinsing the SPE with TRIS-T followed by TRIS. To perform analysis of the serum containing IgG anti-S, the sample was diluted by a 1:10 factor in a TRIS solution containing 45 µg/mL anti-Hu-IgG-AP. The obtained solution was drop-casted for 1 h on the surface of the modified SWCNT-SPE. At the end of the incubation, a washing step was performed with TRIS-T followed by TRIS. Subsequently, the voltammetric readout was carried out.

### 2.7. Validation of SARS-CoV-2 Anti-N and Anti-S Immunosensors

The analytical performance of the immunosensors was assessed in the human serum matrix. For this purpose, sera from the pre-pandemic period were spiked with commercial monoclonal anti-N antibodies in the case of the anti-N immunosensor. Concerning the anti-S immunosensor, a 20,000 BAU/mL anti-S WS [20] was gradually diluted in the same pre-pandemic matrix up to 500 BAU/mL. Firstly, validation of the immunosensors developed for SARS-CoV-2 anti-N and anti-S antibodies detection and quantification was performed, according to the Eurachem Guide [31]. For this purpose, LOD, LOQ, linearity and precision were calculated, performing three replicated measurements for each level explored. As for LOD and LOQ, 10 replicate measurements of blank samples, i.e., matrices containing no detectable analyte, were carried out; LOD was calculated as 3·*s*_0_/√*n* and LOQ as 10·*s*_0_/√*n*, where *s*_0_ is the blank standard deviation and *n* is the number of replicate measurements. Concerning the evaluation of linearity, the regression residuals were calculated, the mean of which over the linearity range was not significantly different from zero (*p* > 0.05). The precision was measured on two levels, i.e., the lower and upper levels of the calibration curve for each immunosensor. The selectivity of the immunosensors was assessed in human serum in terms of the matrix effect, as discussed in Section 3.3. The evaluation of both of the diagnostic tests was carried out in human COVID-19 serum samples from infected subjects provided by the OSR. We referred to the diagnostic guidelines for epidemiological data [23] for the validation of the immunosensors with clinical specimens in terms of sensitivity, specificity and accuracy.

## 3. Results and Discussion

### 3.1. Immunosensors Setup

In the present study, two immunosensors were developed and validated for the detection of anti-SARS-CoV-2 anti-N and anti-S. To tackle this issue, we developed an immunoaffinity assay for both of the analytes, consisting of a first modification of the sensing surface with the receptor antigen, followed by the simultaneous incubation of the target antibody, which specifically interacts with the bound protein, and the secondary antibody conjugated to the enzyme AP. The protein receptors were immobilized on the surface of commercially available disposable screen-printed electrodes with different electrode substrates, e.g., gold-nanoparticles (GNP-SPEs), single-walled carbon nanotubes (SWCNT-SPEs) or a combination of the two nanomaterials (SWCNT/GNP-SPEs). The GNP-SPEs allow for the direct chemisorption of the proteins through the interactions of the cysteine residues with the gold surface, via the well-known formation of a Au–thiolate bond [32], while the SWCNT-SPEs natively bear carboxyl groups which can be activated (e.g., with EDC and NHS) to carry out a coupling reaction with the amine moieties of the receptor antigen [33]. The detection of the anti-N antibodies was carried out, using the N protein as capture antigen, while for the detection of the anti-S antibodies two receptors were tested, namely S1 protein, which is a subunit of the S protein, and RBD-Fc protein, in which the RBD of the SARS-CoV-2 S protein is conjugated to a mouse Fc moiety, allowing immobilization via a rabbit-anti-mouse (RAM) secondary antibody. For the latter we have chosen an indirect attachment on the electrode surface to avoid partial denaturation and the consequent structural destruction of the antigen epitopes often observed when directly immobilizing small protein targets. Different approaches were investigated to ensure the proper immobilization of the antigens on the nanomaterials embedded in the working electrodes of SPEs. In more detail, for the N protein, a functionalization of the SWCNT/GNP-SPEs was carried out through direct chemisorption on GNP, while for RBD-Fc the immobilization of SPEs occurred using a RAM secondary antibody, which, in turn, was immobilized covalently via the EDC/NHS-promoted coupling reaction involving the carboxyl functionalities of the SWCNT-SPEs. As for the S1, direct covalent immobilization was performed on activated the SWCNT-SPEs.

**Figure 2:**
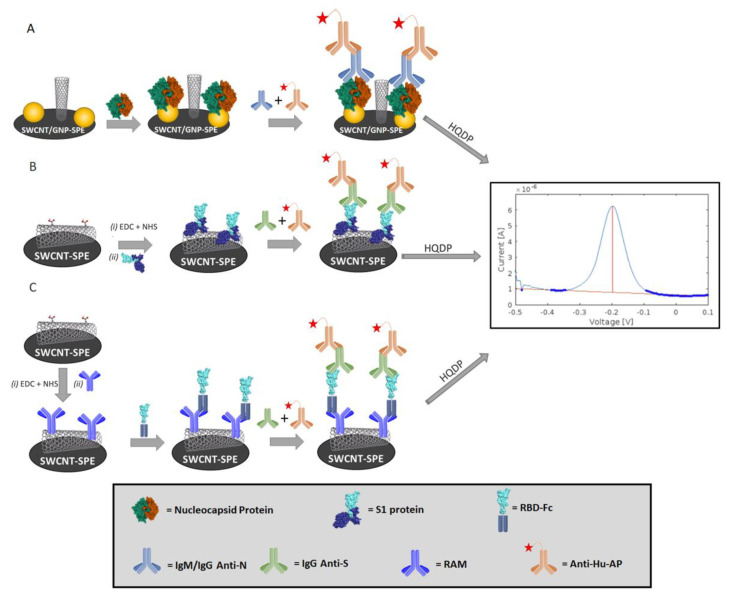
Protocols used for the development of electrochemical immunosensors based on: (**A**) N protein for determination of anti-N antibodies; (**B**) S1; and (**C**) RBD-Fc proteins for determination of anti-S antibodies.

The format of the assays consists of an antigen/antibody/secondary antibody immunochemical setup. After the modification of the sensing surface with receptor antigens, a mixture of the target human antibodies and anti-Hu-AP was incubated on the immunosensor surface. In order to discriminate between the IgG and IgM isotypes, anti-Hu-IgG-AP or anti-Hu-IgM-AP were used as the secondary reading antibodies. The protocols of the proposed electrochemical immunosensors are illustrated in Figure 2. It is worth pointing out that, in the proposed immunosensors, the analysis is carried out through a simple single step where the serum specimen, mixed with the anti-Hu-AP solution, is incubated on the sensor surface for 1 h, thus making the approach time-effective and suitable for PoCT and testing in non-hospital environments. Furthermore, the analysis performed on the portable IoT-Wi-Fi device allows for the real-time sharing of the results through a cloud-based service.

### 3.2. Receptor Antigen Immobilization Methods and Chemistries

**Figure 3:**
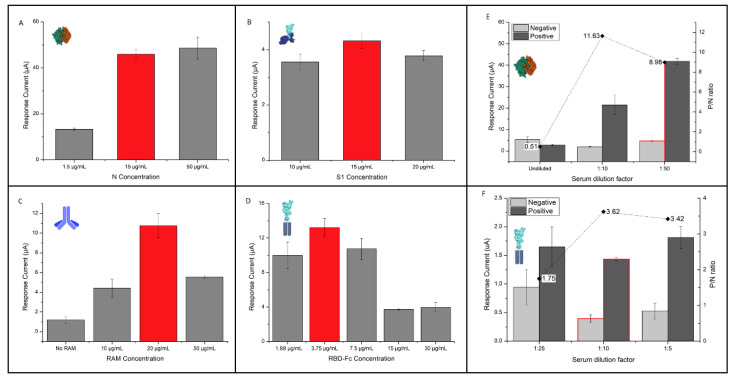
(**A**–**D**): effect of antigen receptor concentration on the current response; (**A**) N protein; (**B**), S1 protein; (**C**) RBD-Fc protein; (**D**) RAM used for proper orientation of RBD-Fc; (**E**,**F**): effect of serum matrix dilution on the current response for immunosensors aimed at the determination of (**E**) anti-N and (**F**) anti-S antibodies; red-contoured columns refer to the selected dilution.

The preliminary tests were carried out in order to assess the best concentration of antigen receptors allowing the maximization of the analytical response. For this purpose, in the case of the N protein, the chemisorption was carried out through direct incubation of the antigen on the SWCNT/GNP-SPEs at concentrations ranging from 1.5 to 50 µg/mL (Figure 3A), while the concentration of the anti-N IgG and anti-Hu-IgG-AP antibodies were fixed at 5 µg/mL and 1 µg/mL, respectively. The best analytical response was observed at 15 μg/mL, whereas no significant improvement was observed by increasing the concentration to 50 μg/mL (*p* > 0.05). As for the S1 protein, its concentration was tested at 10, 15 and 20 µg/mL in the presence of the anti-S1-IgG monoclonal antibodies (5 µg/mL) and anti-Hu-IgG-AP (2 µg/mL) on the SWCNT-SPEs (Figure 3B). The best response was observed for the intermediated level (15 µg/mL), which was then selected for the execution of the experiments.

For the functionalization of the SWCNT-SPEs with RBD-Fc, a preliminary evaluation of the RAM concentration (10-50 μg/mL) to be used for the immobilization of the receptor antigen, fixed at 7.5 µg/mL, was performed. The direct immobilization of RBD-Fc in the absence of RAM (Figure 3C) was also considered. An overall higher response was observed using RAM, confirming the proper orientation of the receptor antigen, achieving the best response at 20 µg/mL of RAM. Under these conditions, the RBD-Fc concentration ranging from 30 to 1.88 µg/mL on the SWCNT-SPEs was evaluated (Figure 3D). The best results were obtained at 3.75 µg/mL, since lower concentrations do not allow the correct addressing of all of the available RAM interaction sites, while at the higher concentrations the signal intensity decreases, probably due to overcrowding of the antibody-binding sites. It should be noted that no signals were obtained using the SWCNT/GNP-SPEs for the immobilization of the S1 or RBD-Fc receptor antigens. This result is indirect evidence of an immobilization issue on the gold electrode substrate, as also supported by a recent study in which, in order to conjugate the SARS-CoV-2 antigens epitopes with gold nanoparticles, a streptavidin/biotin strategy was used to develop a nano-sensing platform [34]. For this reason, the SARS-CoV-2 anti-S immunosensors were implemented on the SWCNT-SPEs.

In order to confirm the immobilization of the antigen receptors for both of the developed immunosensors, we recorded cyclic voltammograms, using ferrocyanide as a redox probe, noting a progressive decrease in the diffusion current during the sequential functionalization of the SPEs (Figure S1, Supplementary Materials).

Finally, we explored the response upon different sample incubation times, ranging from 30 to 90 min, under the best conditions found for 53h RAM and RBD-Fc concentrations. The best compromise in terms of signal intensity and precision was found by operating with an incubation time of 1 h (Figure S2, Supplementary Materials)

### 3.3. Effect of Serum Matrix

After establishing the conditions for the modification of the sensor surface with each receptor, the effect of the serum matrix was assessed, evaluating the response obtained by testing both anti-N and anti-S in human serum. Since the analytical signal is generated by the anti-Hu-AP specific for IgG or IgM, as human serum is rich in these immunoglobulins, interference is likely to occur. As a result, a limited number of anti-Hu-AP will be available to interact with the target antibodies, thus inducing possible false negative results. This effect is especially critical for the IgG antibody isotype, since the human serum samples physiologically contain higher concentrations of these immunoglobulins. To overcome this matrix effect, we decided both to dilute the sample and to increase the anti-Hu-AP secondary antibody concentration in order to maximize the positive signal obtained in the presence of target antibodies, while keeping the negative signal acquired in the absence of target antibodies as low as possible. To establish the appropriate matrix dilution factor for the anti-N immunoassay, the positive and negative signals were acquired by analyzing both the undiluted serum and matrix diluted by a factor of 1:10 and 1:50, while maintaining the anti-Hu-IgG-AP at 20 µg/mL and anti-N-IgG at 5 µg/mL (Figure 3E). Under these conditions, the highest positive/negative (P/N) ratio was obtained with a 1:10 dilution; however, compared to the 1:50 dilution, the positive signal of the 1:10 dilution shows approximately half the peak intensity and high data dispersion (RSD > 10%). Therefore, a serum dilution factor of 1:50 was applied for the subsequent measurements. As for the anti-Hu-AP concentration, the results obtained at 10 and 20 µg/mL were compared both for IgG (Figure S3A, Supplementary Materials) and for IgM (Figure S3B, Supplementary Materials), showing a higher P/N ratio for the lowest concentration. Regarding the anti-S immunosensor, since the serologic testing was also devised with the aim of quantifying the antibody titer, we used the WS previously described in the introduction to assess the experimental conditions leading to the best P/N ratio. However, critical issues concerning the S1 receptor were observed in the presence of the serum matrix. In fact, it was observed that the response given by the negative samples (pre-pandemic sera) was more intense than that obtained by testing the same negative samples in the absence of the receptor (Figure S4, Supplementary Materials). This suggests that the S1 receptor cross-interacts with the species present in the serum samples, giving rise to non-specific signals. For this reason, the development of the immunosensor exploited the S1 subunit’s receptor-binding domain (RBD)-Fc to capture the target antibodies. On the basis of the results obtained for the anti-N assay, different dilution factors of the sample were tested, namely 1:25, 1:10 and 1:5 (Figure 3F) using a pooled working standard (WS) from vaccinated subjects [20] with an anti-S-IgG concentration of 511 BAU/mL, estimated by the OSR through chemiluminescence immunoassay, and the pooled human sera from the pre-pandemic period as negative samples. Comparing the P/N ratios obtained from the WS, the best results were observed using a dilution factor of 1:10. To further increase the P/N ratio, the concentration of the anti-Hu-AP secondary antibody was considered, exploring levels of 20, 30, 40 and 45 µg/mL (Figure S3C, Supplementary Materials). The P/N ratio showed an increasing trend with increasing anti-Hu-AP concentration, therefore the value chosen for subsequent studies was 45 µg/mL.

Using the RBD-Fc receptor, there was no evidence of non-specific interactions with the serum matrix, showing a comparable response when negative samples, i.e., pre-pandemic sera, were tested in the presence or absence of the receptor (Figure S4, Supplementary Materials). Comparing the two receptor antigens tested for anti-S determination, no significant difference (*p* > 0.05) between the 511 AU/mL level and the negative sera was observed when using the S1 receptor, whereas the tests performed with RBD-Fc led to a P/N of approximately four, proving a better sensitivity of the RBD-Fc receptor compared to the S1, which was then discarded.

### 3.4. Linearity and Sensitivity Assessment of SARS-CoV-2 Anti-N and Anti-S Immunosensors

**Figure 4:**
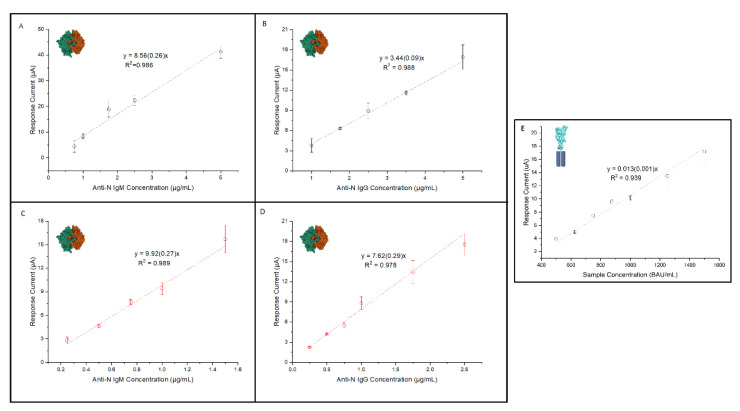
(**A**–**D**): calibration lines obtained in human serum for the determination of (**A**) anti-N IgG on GNP-SPEs; (**B**) anti-N IgG on SWCNT/GNP-SPEs; (**C**) anti-N IgM on GNP-SPEs; (**D**) anti-N IgM on SWCNT/GNP-SPEs; (**E**): calibration line obtained from properly diluted working standard for the determination of anti-S IgG on SWCNT-SPEs.

The linearity of the anti N-immunosensors for the detection of IgG and IgM was investigated by using different electrode substrates. In particular, when using GNP-SPEs and exploring concentrations of anti-N IgM antibodies from 0.5 to 10 μg/mL, a linear response from 0.75 to 5 μg/mL was observed, with a corresponding LOD and LOQ of 0.18 and 0.56 μg/mL, respectively (Figure 4A). As for the anti-N IgG antibodies, a concentration range from 0.75 to 10 μg/mL was explored, showing a linear response between 1 and 5 μg/mL, with a LOD and LOQ of 0.21 and 0.6, respectively (Figure 4B).

Similar experiments were carried out on the SWCNT/GNP-SPEs to evaluate the performance improvement guaranteed by the combination of two nanomaterials, since the GNPs allow the immobilization of the receptor antigen, whereas SWCNTs increase the active surface of the electrode and improve the electron transfer efficiency. Using these high-performance electrode substrates for the detection of the anti-N IgM antibodies, the response was investigated from 0.25 to 1.75 µg/mL (Figure 4C), showing linearity between 0.25 and 1.5 µg/mL. Better results in terms of the LOD (0.05 µg/mL) and LOQ (0.16 µg/mL) were achieved for IgM determination compared to the GNP-SPEs. Furthermore, good precision was observed at the lower and upper level of the calibration curve, resulting in RSD values of 5% at 0.25 µg/mL and 8% at 1.5 µg/mL. As for the anti-N IgG, we tested concentrations ranging from 0.25 to 5 µg/mL (Figure 4D), observing a linear response from 0.25 to 2.5 µg/mL with LOD and LOQ values of 0.06 and 0.19 µg/mL, respectively. Precision assessment resulted in RSD values of 4% at 0.25 µg/mL and 9% at 2.5 µg/mL. These findings show that the use of the SWCNT/GNP-SPEs increases the immunosensor sensitivity, in particular for the determination of IgG antibodies, where the presence of SWCNTs has resulted in a sensitivity more than double that observed on the GNP-SPEs. In our opinion, the improvement in the performance of the immunosensor, also attested to by the shift of the LOD and LOQ towards lower values, compensates for the slight cost difference of using the more expensive SWCNT/GNP-SPEs. These electrode substrates were then selected for the determination of both of the isotypes of anti-N antibodies.

To assess the linearity of the immunosensor based on RBD-Fc receptor, different levels were explored by testing the anti-S-IgG ranging from 500 to 2000 BAU/mL (Figure 4E). The tests were performed on the SWCNT-SPEs using the sample dilution factor discussed in Section 3.3. A linear response was observed in the 500 to 1500 BAU/mL range, resulting in a LOD and LOQ of 210 and 233 BAU/mL, respectively. Precision assessed at the lower and upper concentration levels of the calibration, i.e., 500 and 1500 BAU/mL, resulted in RSD values of 9% and 7%, respectively. Taking into account the low presence of IgM positive clinical samples, and that IgG are the most relevant antibodies for the evaluation of the immune response induced by vaccination, the study continued on the detection of the anti-S-IgG antibody isotype.

### 3.5. Performance of the Immunosensors in Clinical Specimens

**Figure 5:**
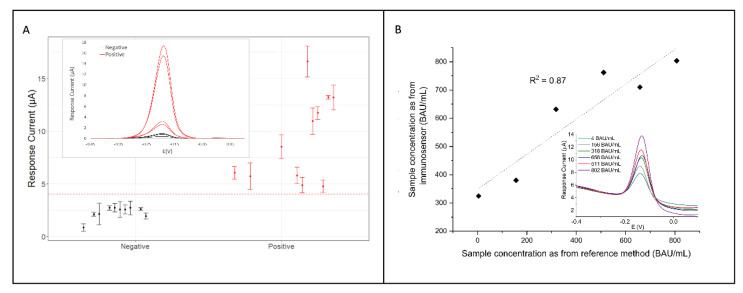
(**A**): scatterplot of current responses acquired analyzing serum specimens containing anti-N IgG antibodies. Inset: DPV voltammograms for selected positive and negative specimens; (**B**): correlation between anti-S IgG concentration in serum specimen estimated by the immunosensor versus the corresponding values obtained by reference Roche Elecsys^®^ chemiluminescence immunoassay. Inset: DPV voltammograms for the selected serum specimens with labels referred to the concentration values assessed using Roche Elecsys^®^.

The developed immunosensors were validated using clinical patient serum samples provided by the OSR and tested with the Roche Elecsys^®^ Anti-SARS-CoV-2 chemiluminescence immunoassay, which was used as the reference method to assess the correct classification of positive/negative results. Regarding the anti-N immunosensor, a total of 21 serum specimens consisting of 10 negatives (anti-N concentration < 1 AU/mL) and 11 positives from SARS-CoV-2 infected patients (anti-N concentration > 1 AU/mL) were analyzed (Figure 5A). Although the limited number of samples tested did not allow a thorough clinical validation of the proposed anti-N immunosensor, it was possible to correctly classify all of the samples analyzed, thus proving excellent performance in terms of sensitivity, specificity and accuracy, reaching almost 100% for all of the parameters evaluated. These findings prove the fitness for purpose of the immunosensors, despite the RSD values, referred to inter-sensor repeatability.

As for the anti-S immunosensor, given the problems posed by obtaining standards at known concentrations, the recovery of the assay was assessed by processing samples obtained by diluting the WS with pre-pandemic human serum. From the undiluted WS (20,000 BAU/mL) we obtained three solutions at concentrations of 600, 800 and 1100 BAU/mL. Our immunosensor reported concentrations of 570 BAU/mL, 854 BAU/mL and 1023 BAU/mL, corresponding to excellent recovery values of 95%, 106% and 93%. As for the analysis of the single-patient serum specimens, six samples from subjects previously infected by SARS-CoV-2 were processed and the anti-S concentrations, calculated using the calibration curve reported in Figure 4E, were compared with the titers obtained at the OSR, using the Roche Elecsys^®^ chemiluminescence immunoassay (Figure 5B).

A good correlation (R^2^ = 0.87) between the two devices was obtained, consistent with the fact that the two devices shared the same protein target (S-protein RBD). It should be noted that there is still a lack of harmonization/standardization among the devices produced by different manufacturers. In this regard, a recent study showed that when comparing devices based on both different technologies and different S-protein targets (i.e., monomeric soluble form, trimeric form, RBD motif) the correlation factors were as low as R^2^ = 0.13 [20]. Overall, these results show that developed immunosensors represent a powerful diagnostic tool for rapid and effective serological tests. Compared to the previously published papers [16,17,18,19], the competitive advantages have demonstrated unique features of the developed biosensors which are based on:

(i) having also dealt with the detection and determination of anti-N antibodies, in addition to anti-S, thus providing more informative results;
(ii) the use of working standards of anti-S from vaccinated subjects cross-calibrated against the international standard from the WHO [20] for the determination of anti-S antibodies in clinical sera of infected patients;
(iii) having demonstrated reliability in real serum samples through a thorough validation of the immunosensors both in serum matrix and clinical samples.

### 3.6. Performance of the Readout Instrumentation

These good analytical results are further enhanced by the adoption of our portable Wi-Fi potentiostat. Given the acquisition parameters described in Section 2.3, our solution results in a total of 342 sampling points. Other portable solutions reported in the literature use AFEs based on COTS components, such as the LMP91000 device from Texas Instruments [35], but they result in an order of magnitude fewer points [29], preventing them from performing quantitative analysis.

The total time needed for each acquisition is equal to 1.5 min when processing is performed onboard, representing another feature of the instrumentation proposed.

An assessment of the power consumption was carried out. The average current of a basic prototype, which was already discussed in a previous work [29], results in 81 μA. Since the stand-by current of the barcode scanner device is 3.5 mA, this contribution becomes overwhelming to estimate the battery life. Considering a compact (34 mm × 41 mm × 4.2 mm) Li-Ion battery with a capacity of 600 mAh, it is possible to get 7 days of operation 24 h a day without recharging the device. It should be noted that in the real context the device has to work for about 8 h a day, hence the autonomy reached by our Wi-Fi-IoT device is very good compared to the state of the art. In the recent paper of Torrente-Rodrıguez et al. [16], a similar wireless device was described with a battery life of 5 h, whereas Ainla et al. [36] devised an open-source “universal wireless electrochemical detector” reaching a battery life of 60 h. During the battery life, the output voltage ranges from 4.2 V (i.e., charging voltage) to 2.8 V (i.e., discharge cut-off voltage) and is stabilized to allow the device to operate properly.

Further, our device, unlike other devices described in the literature and commercially available [19,37,38,39,40], benefits from a Wi-Fi portable potentiostat connected to a cloud and a web service for accessible and intuitive user interaction, sharing data between end-users/caregivers, and independent of external devices (PC, tablet or smartphone) and custom apps. In the system here proposed, the IoT portable potentiostat is connected to the ThingSpeak cloud service, which is a web solution for IoT projects that allows data collection in the cloud, and their processing and analysis exploiting MATLAB capabilities [41]. This solution is not limiting, as other clouds with analytics features may be used. Thanks to the adoption of the Wi-Fi protocol the device can be easily inserted into a network by including other devices in a IoT vision.

These features, combined with the ease of use of the portable device, allow for widespread screening programs.

## 4. Conclusions

In the present work, smart high-performance immunosensors for PoCT serological tests to detect the presence of anti-SARS-CoV-2 antibodies in serum were successfully developed to meet the growing demand for fast, easy-to-use and accurate diagnostics to facilitate testing outside the laboratory setting.

The applicability of the developed immunosensors to the detection and quantification of anti-N and anti-S in serum samples from both vaccinated and SARS-CoV-2 infected patients was demonstrated. The immunosensors showed excellent performance in terms of sensitivity, specificity and accuracy, as well as a reliable quantitative determination of antibodies that can be attained in a single step. In fact, the execution protocol of the electrochemical immunoassay is simple and fast, requiring only the sample dilution in the buffer containing all of the reagents and a single one-hour incubation step.

The high portability and versatility features give the method excellent potentiality for application in screening programs aimed at assessing the degree of COVID-19 immunization of the population.

The design of a PCB including both analog and digital sections is planned in the near future, in order to obtain even smaller dimensions and to enhance device portability. Furthermore, the integration of portable power sources will improve the functionality of the test; this function being especially useful in environments where electricity is not available.

The proposed system combines the unique features of immunochromatographic strip-tests (simple and single-step sample incubation) with the remarkably superior performance of electrochemical immunosensors. Furthermore, the system allows the quantitative determination of the antibody titer and the simultaneous storage of the result in epidemiological databases; such characteristics are not obtainable with the LFIA strip tests.

## Data Availability

The data are available upon reasonable request from the corresponding author.

## Supplementary Materials

The following supporting information can be downloaded at: https://www.mdpi.com/article/10.3390/s22145463/s1, Figure S1: Cyclic voltammetry (CV) characterization of the modified SPEs; Figure S2: Effect of sample incubation time; Figure S3: Effect of AP-conjugated secondary antibodies; Figure S4: Interference studies for the S1 and RBD-Fc receptors in serum matrix.
