# Supplementary material for: Smart Immunosensors for Point-of-Care Serological Tests Aimed at Assessing Natural or Vaccine-Induced SARS-CoV-2 Immunity
Source: Sensors (Basel). 2022 Jul 21;22(14):5463. doi: 10.3390/s22145463 (PMC9325165; doi:10.3390/s22145463)
Supplement: Supplementary file 1 [file sensors-22-05463-s001.zip › sensors-1798327-supplementary.pdf]

# Smart immunosensors for point-of-care serological tests aimed at assessing natural or vaccine-induced SARS-CoV-2 immunity

**Simone Fortunati[1], Marco Giannetto[1,*], Chiara Giliberti[1], Angelo Bolchi[1], Davide Ferrari[1], Massimo Locatelli[2], Valentina Bianchi[3], Andrea Boni[3], Ilaria De Munari[3] and Maria Careri[1,*]**

[1] Dipartimento di Scienze Chimiche, della Vita e della Sostenibilità Ambientale, Università di Parma, Parco Area delle Scienze 17/A, 43124 Parma, Italy; simone.fortunati@unipr.it; marco.giannetto@unipr.it; chiara.giliberti@unipr.it; angelo.bolchi@unipr.it; davide.ferrari@unipr.it; maria.careri@unipr.it

[2] IRCCS Ospedale San Raffaele, Via Olgettina 60, 20132 Milano, Italy; locatelli.massimo@hsr.it

[3] Dipartimento di Ingegneria e Architettura, Università di Parma, Parco Area delle Scienze 181/A, 43124 Parma, Italy; valentina.bianchi@unipr.it; andrea.boni@unipr.it; ilaria.demunari@unipr.it

*Correspondence: marco.giannetto@unipr.it; maria.careri@unipr.it

# Supplementary material

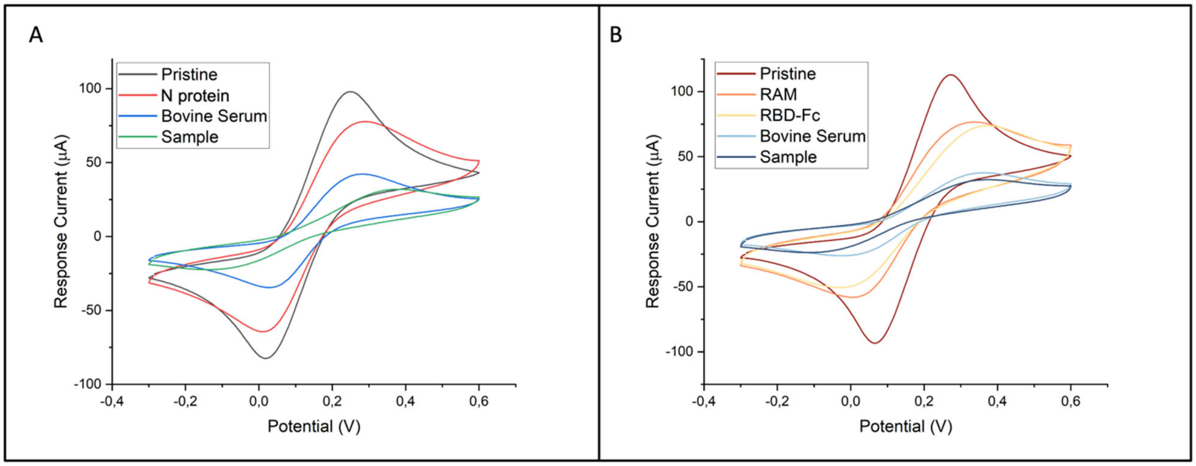


**Figure S1**. Cyclic voltammograms acquired after each functionalization step for (A) anti-N immunosensor on SWCNT/GNP-SPEs and (B) anti-S immunosensor on SWCNT-SPEs using ferrocyanide as redox probe for the characterization of SPEs functionalization.

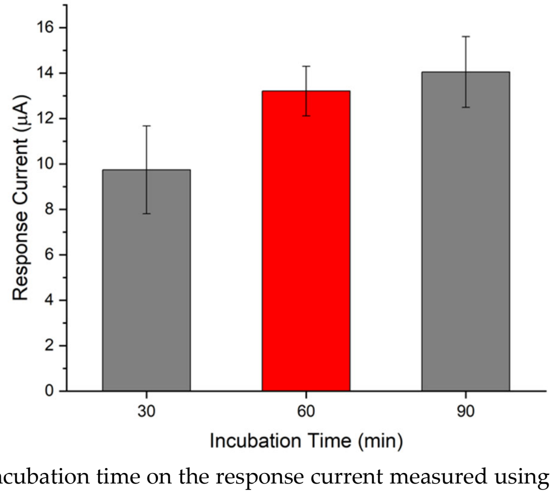


**Figure S2**. Effect of sample incubation time on the response current measured using the anti-S immunosensor based on RBD-Fc.

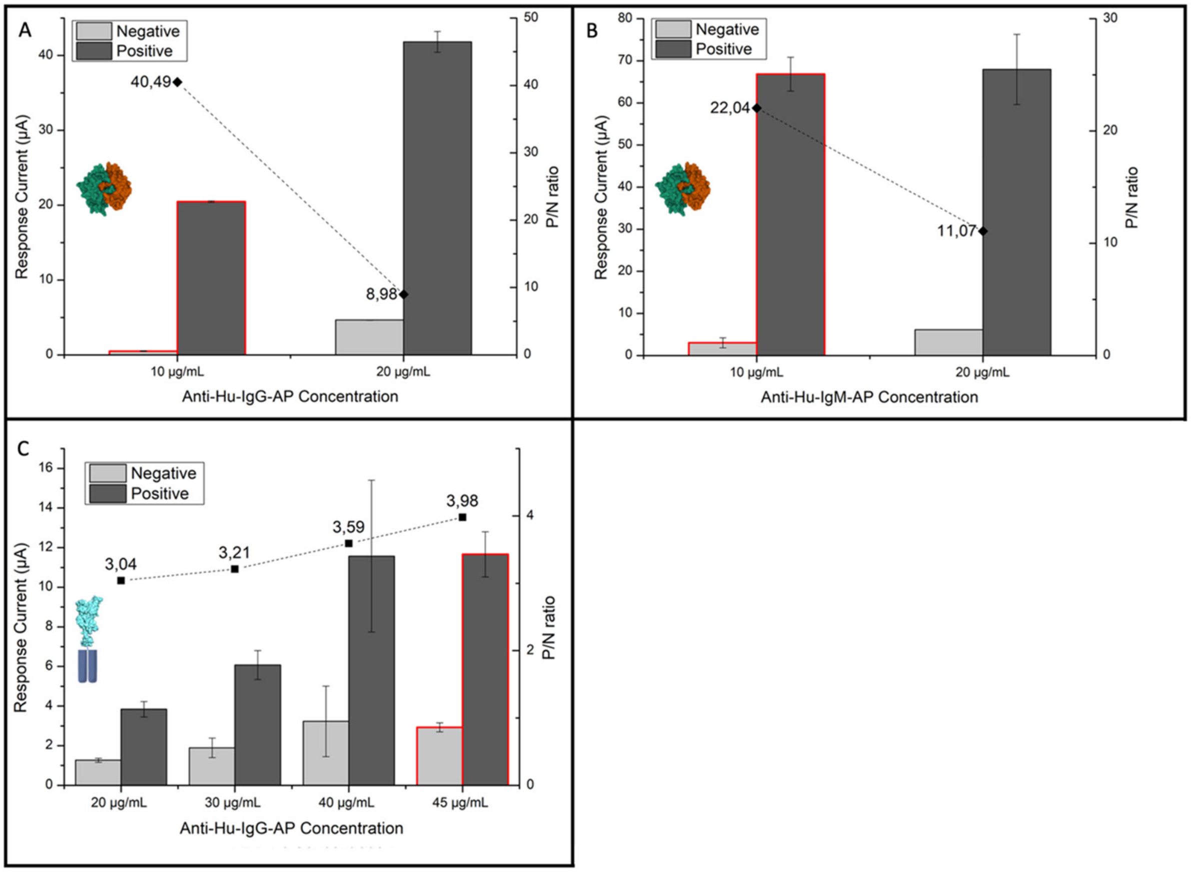


**Figure S3**. Effect of AP-conjugated secondary antibodies concentration on the response current of (A) anti-N IgG, (B) anti-N IgM, (C) anti-S IgG; red-contoured columns refer to the selected concentration.

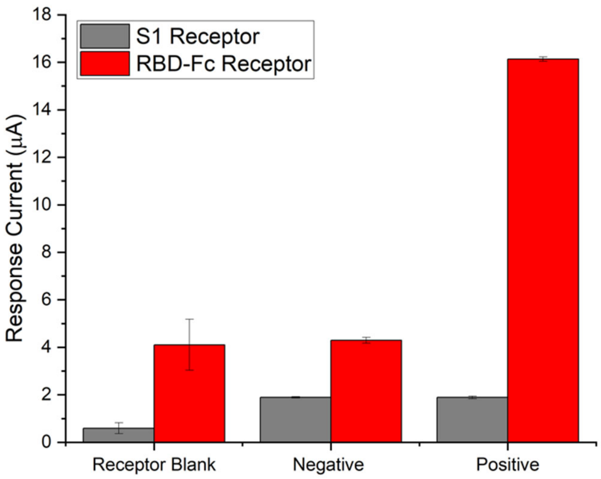


**Figure S4**. Comparison of response current observed by incubating negative samples in the presence and absence of S1 and RBD-Fc receptors and positive sample in the presence of receptors.
